# SWATH-MS analysis of plasma proteins among Indian HIV-1 infected patients

**DOI:** 10.6026/97320630019392

**Published:** 2023-04-30

**Authors:** Sushanta Kumar Barik, Srikanth Prasad Tripathy, Deepa Bisht, Praveen Singh, Rahul Chakraborty, Shripad A Patil, Tej Pal Singh, Deepika Varshney, Srikanta Jena, Keshar Kunja Mohanty

**Affiliations:** 1 ICMR-National JALMA Institute for Leprosy and Other Mycobacterial Diseases, Agra, Uttar-Pradesh, India-282004; 2 ICMR-National Institute for Research in Tuberculosis, Chetpet, Chennai, Tamil Nadu, India-600031; 3 CSIR-Institute of Genomics and Integrative Biology, CSIR, New Delhi-110025; 4 Sarojini Naidu Medical College, Agra, Uttar-Pradesh, India-282003; 5 Ravenshaw University, Odisha, Cuttack, India - 753003

**Keywords:** SWATH-MS, HIV-1, AIDS, ART, NRTI, NNRTI

## Abstract

The identification and characterization of plasma proteins in drug resistant and drug sensitive in HIV-1 infected/AIDS patients were carried out using the SWATH-MS protocol. In total, 204 proteins were identified and quantified, 57 proteins were differentially expressed, out of which 25 proteins were down regulated and 32 proteins were up regulated in drug resistant patients. Six proteins such as complement C4-A, immunoglobulin heavy variable 1-2, carboxylic ester hydrolase, fibulin-1, immunoglobulin lambda constant7, secreted phosphoprotein 24 were differentially expressed in individuals with drug resistant HIV as compared to individuals with drug sensitive HIV. Gene ontology of 57 differentially expressed proteins was analysed and documented.

## Background:

The analysis of HIV-1 infected human plasma is an attractive medium with which differentially expressed proteins could be identified in certain disease states. Proteomic techniques are widely used globally to identify differentially expressed plasma proteins in response to HIV-1 infection. Sequential window acquisition of all theoretical mass spectra (SWATH-MS) is a novel technique conceptualized with the protein library and the individual protein was identified and quantified for biomarker discovery [1, 2]. Therefore, it is of interest to characterize and quantify the plasma proteins of drug-resistant and drug-respondent patients over 6 years of first-line ART by SWATH-MS.

## Materials and Methods:

## Materials:

Ammonium formate, formic acid, dithiothreitol (DTT) and iodoacetamide (IAA) were procured from Sigma, USA. Modified trypsin (sequencing grade) was procured from Promega, USA. Polysulfoethyl SCX cartridge (5 micron, 300 Å bead) with cartridge holder was procured from Sciex, USA. Acetonitrile, Liquid phase-chromatography-mass spectroscopy grade water, was procured from Biosolve (Lorraine, France). Analytical grade chemicals were used for this study.

## Study design and Study participants:

The study focused on patients administered six first-line ART such as ZLE, ZLN, TLE, TLN, SLE and SLN patients and they were enrolled in the anti-retroviral therapy centre, Sarojini Naidu Medical College, Agra, India from December 2009 to November 2016 as per the treatment guidelines provided by NACO, Govt. of India and the ethical guidelines formulated by the ICMR, Government of India. The age of the six patients varied from 32 years to 43 years. A patient information leaflet was used for data collection [3]. Ten ml of blood was collected from each patient whose CD4+ count was <350 cells/µl. The plasma was separated after centrifuging at 2000g for 10 minutes. The separated plasma was stored at -800 C for viral load and genotyping was carried out at the National Institute for Research in Tuberculosis, ICMR, Chennai, Tamilnadu, India. The viral load was estimated in all six plasma samples. Among them, 3 patients had a higher viral load >1000 copies/ml (drug resistant HIV infection) and 3 patients had a lower viral load <1000 copies/ml to target not detected level (drug sensitive HIV infection. Three drug-resistant patients (>1000 copies/ml) were considered for genotyping.

## Ethics committee and informed consent:

The study has been approved by the Institutional Ethics Committee of the ICMR- National JALMA Institute for Leprosy and Other Mycobacterial Diseases. The written informed consent was obtained from each study participant before enrolling in the study and collection of the blood samples [3].

## Genotyping:

The genotyping was performed for the samples with a viral load ≥1000 copies/ml at the ICMR-National Institute for Research in Tuberculosis, Chennai, by using the WHO dried blood spot protocol 2010 [4]. The details of the PCR primers and reaction conditions were reported [5].

## Sample preparation for proteomics analysis:

100µl plasma was inactivated by heating at 56°C for 30 min [6]. From this, 60µl plasma was used for albumin and globulin depletion using an Aurum serum mini kit (BioRad, USA). The six depleted plasma samples were used for SWATH-MS analysis. Protein estimation was done on the depleted plasma protein samples using the Bradford assay (Sigma-Aldrich, USA).

## SWATH-MS protocol for plasma proteins:

Reduction, alkylation, and trypsin digestion: For SWATH run, 10µg of proteins from each sample were reduced with 25mM DTT for 25 min at 56°C, followed by alkylation using 55mM IAA at room temperature for 15-20 min in the dark, and trypsin digestion for 18 h at 37°C. Tryptic peptides were vacuum-dried in a vacuum concentrator.

## LC-MS/MS data acquisition:

Library generation for SWATH analysis: For generation of the spectral ion library in data-dependent acquisition mode, 300 µg of protein obtained by pooling of plasma from the six drug resistant and drug respondent patients were digested using trypsin and were fractionated into 8 fractions by cation exchange (SCX) chromatography using a SCX cartridge (5 micron, 300 Å bead from AB Sciex, USA) and a step-gradient of increasing concentration of ammonium formate buffer (35 mM, 50 mM, 75 mM, 100 mM, 125 mM, 150 mM, 250 mM and 350 mM ammonium formate, 30% v/v ACN and 0.1% formic acid; pH = 2.9). Peptides from each of these fractions were cleaned-up using C18 ZipTip (Millipore, USA). Each fraction was then analysed on a quadrupole-TOF hybrid mass spectrometer (TripleTOF 6600, Sciex, USA) coupled to a nano-LC system (Eksigent NanoLC-425). 4 µg of these peptides were loaded on a trap-column (ChromXP C18CL 5µm 120Å, Eksigent) and desalted at a flow rate of 10 µl per minute for 10 min. Peptides were then separated on a reverse phase C18 analytical column (ChromXP C18, 3µm 120 Å, Eksigent) using buffer A (98 % water with 0.1 % formic acid and 2 % acetonitrile) and buffer B (98 % acetonitrile with 0.1 % formic acid and 2 % water) and the following gradient: buffer B was increased gradually from 3% to 25% in the first 68 min. It was increased to 35% solvent B in the next 5 minutes, In the next 2-minute buffer B was ramped up to 80% and held at the same concentration for next 3 minutes. Buffer B was swiftly brought to an initial 3% concentration in the next 1 min and held at the same concentration till the end of 87 minutes gradient. The optimized source parameters were as follows: the ion spray voltage was to 5.5 KV, curtain gas was set at 25 psi, nebulizer gas was set at 10 psi and source temperature was set at 200°C. For DDA, a 1.8 s instrument cycle was repeated in high sensitivity mode throughout the entire gradient, consisting of a full scan MS spectrum (400-1250 m/z) with an accumulated time of 0.25s, followed by 30 MS/MS experiments (100-1500 m/z) with 0.050 s accumulation time each, on MS precursors with charge state 2+ to 5+ exceeding a 150-cps threshold. Rolling collision energy was used and the former target ions were excluded for 15 seconds. For the DIA-SWATH run, instrument and chromatographic conditions were identical to the DDA run for library preparation. 100 precursor isolation windows were defined based on precursor m/z frequencies in the DDA runs, with a minimum window width of 5 m/z using the SWATH Variable Window Calculator (Sciex). The accumulation time was set to 0.25 sec for the MS scan (400-1250 m/z) and 0.025 s for the MS/MS scans (100-1500 m/z) respectively. Rolling collision energies were applied for each window based on the m/z range of each SWATH and a charge 2+ ion, with a collision energy spread of five. The total cycle time was 2.84 s.

## Database searches and peak extraction:

The spectral ion library is a merge search for 8 DDA runs and analysis in protein pilot software v5.0.1 (Sciex, USA) with the paragon algorithm, to obtain protein identities. The parameters were set as follows: cysteine alkylation-IAA, digestion-trypsin. The search effort was set to 'thorough ID' and false discovery rate (FDR) analysis was enabled. Proteins identified with a 5% FDR were considered. The search was carried out against the UniProt database containing 20,350 human proteins. The result (.group) file, thus generated served as the spectral ion library. SWATH generated data processing was performed using the SWATH Acquisition Microapp 2.0.1 in PeakView 2.1 Software. Protein Pilot search result file (.group) was imported with 251 specified proteins, and shared peptides were excluded. Retention time alignment was performed using peptides from abundant proteins. The processing settings for peak extraction were set as: 10 peptides per protein, 5 transitions per peptide, >95% peptide confidence threshold, 5%FDR and exclude modified peptides. The XIC extraction window was set to 85 min with a 50 ppm XIC width. All information was exported in the form of Marker View (.mrkw) file. In the SWATH-MS analysis, the following steps were carried out: reduction, alkylation and trypsin digestion, Library generation for SWATH analysis, data searches and peak extraction using the published protocol of Ghose et al, 2019, Basak et al, 2015 [7, 8].

## Statistical analysis:

The processed mrkvw files from PeakView were then loaded onto MarkerView (version 1.2.1, AB Sciex) and the total area sum normalisation was performed, protein peak areas were then exported to excel where statistical analysis (Student's t-test) was performed. Proteins with a 1.5-fold increase or decrease (p-value < 0.05) were taken as differential proteins.

## Results:

## Genotyping data:

Blood samples from three drug resistant patients having a viral load of 9983 copies/ml, 12344 copies/ml, 36177 copies/ml respectively and three drug sensitive patients‘ blood samples with a viral load of <40 copies/ml, were analysed for SWATH MS study. The NRTIs and NNRTIs associated mutations M41L, T215Y, M184V, T215F, and Y188L, G190A were identified in three drug-resistant patients' samples.

## Proteomics data:

**Figure 1:**
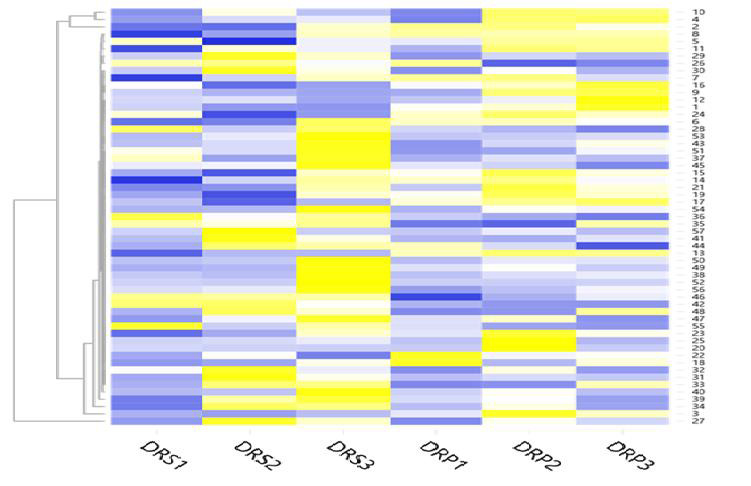
Heat map of the differentially expressed down regulated and up regulated proteins DRS represent Drug resistant and DRP represents Drug respondent. The expression of each protein is based on Z score range from -2 (blue colour) to 2 (Yellow colour).

Protein folds change analysis of HIV drug-resistant and HIV drug- sensitive patients: Retention time (RT) calibration of peptides and peak area extraction was performed in the SWATH micro app of PeakView and data normalization was done in MarkerView. Peak areas were then exported to excel and then statistical analysis was performed. The data were normalized using total area sum normalization. The normalized fold change over drug resistant to drug sensitive HIV was calculated and >1.5 value was considered as significantly upregulated proteins and <0.67 value for significantly downregulated proteins. Finally, a student t-test was done to compare between drug resistant and drug sensitive patients for each detected protein. A value of p<0.05 was considered significant. Thirty-two proteins were identified as upregulated and twenty-five proteins were identified as down regulated in the HIV drug resistant patients. The heat map of the differentially expressed proteins was designed based on the Z score [9]. The expression profile of each differentially expressed protein is presented in the heat map in Figure 1.

## Data analyses:

**Figure 2:**
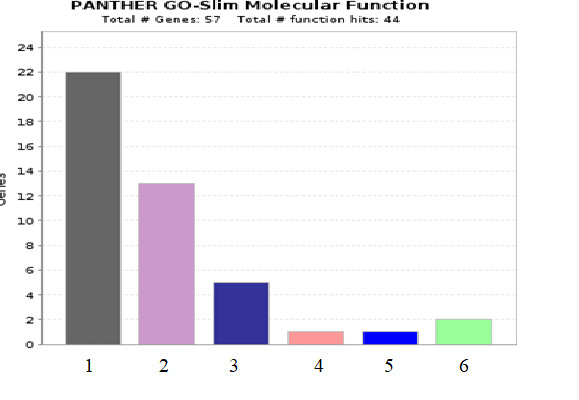
Molecular functions of the differential expressed proteins-PANTHER -gene analyst.

**Figure 3:**
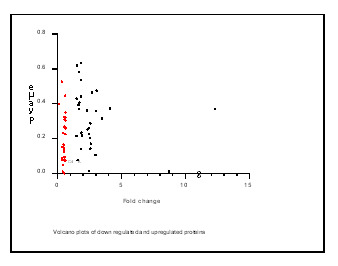
Volcano plots of down -regulated and up-regulated proteins

All 57-protein gene symbol and ID were retrieved through Uniprot (www.uniprot.org). The details of the molecular functions of the 57 differentially expressed proteins in the HIV drug resistant patients was analysed using PANTHER-gene analyst [10]. The molecular functions of the differentially expressed proteins are presented in the Figure 2. Analysing the panther pathway component list and panther pathway suggested that a few plasma proteins were involved in important pathways like the FAS signalling pathway, blood coagulation pathway, cadherin signalling pathway, B-cell activation, Wnt signalling pathway, plasminogen activating cascade, integrin signalling pathway, nicotinic acetyl choline receptor signalling pathway, muscarinic acetyl choline receptor 1 and 3 signalling pathway, muscarinic acetyl choline receptor 2 and 4 signalling pathway, Alzheimer disease-presenilin pathway and CCKR signalling map of HIV-1 patients. The major identified 22 plasma proteins in HIV-1 patients were involved in the binding activity in the panther molecular function list. The Reactome pathway database (https://reactome.org/) was used [11]. The 25 most relevant pathways were inter connected with these 57 differentially expressed proteins in the plasma of drug resistant patients. Gene enrichment analysis of 57 differential expressed proteins encoded by genes of the drug resistant patients was performed by Fun Rich (http://www.funrich.org/) [12]. In the Biological pathway, a set of genes (29.41%) was involved in homeostasis, signalling events mediated by VEGFR1 and VEGFR2, VEGF and VEGFR signalling network, Sphingosine 1-phosphate (S1P) pathway, integrin family cell surface interactions and FAS (CD95) signalling pathway. In biological processes, a set of genes was involved in (23.33%) cell growth and maintenance (20.0%) and immune response (16.66%). Regarding the molecular function, a set of genes were involved in protease inhibitor activity (13.33%) and transporter activity (10.0%). The site of expression of a set of genes (93.54%) was in the plasma of drug resistant patients. The transcription factor HNF4A regulates the expression of a set of genes (47.82%) in the plasma of drug resistant patients. The protein domain of a set of genes (72.41%) acts as signal peptide. In the clinical phenotype, a set of genes (90.90%) was found to be involved in autosomal dominant inherited diseases in the drug resistant patients. The differentially expressed proteins are presented in the volcano plot in the Figure 3. The volcano plot was made using Graph Pad prism 8.0 (Graph pad prism, USA). All 57 differentially expressed proteins encoded by genes of the drug resistant patients were analysed using the Kyoto Encyclopaedia of Genes and Genomes (KEGG) (https://www. genome.jp/) [13]. In the KEGG pathway, these 57 proteins were involved in 28 pathways, interacted with two networks, and expressed in 26 diseases.

## Protein-protein interaction network:

**Figure 4:**
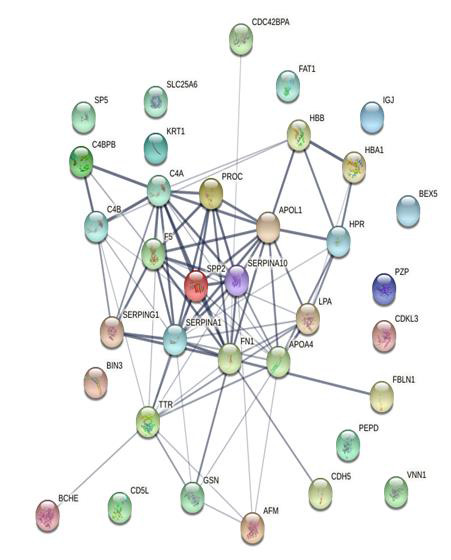
Protein-protein interaction network

Protein-protein interactions among all the differentially expressed plasma proteins were analysed using STRING v.11 (STRING) [14]. Protein-protein interactions were investigated and link was shown with the functional partners (Figure 4).

## Gene ontology of statistically differentially expressed proteins:

The statistically significantly differentially expressed three proteins, such as complement C4-A (fold change=0.45, P=0.05), immunoglobulin heavy variable 1-2 (fold change=0.53, P=0.005), carboxylic ester hydrolase (fold change=0.43, P=0.01) were down regulated and the three proteins fibulin-1(fold change=1.82, P=0.03), immunoglobulin lambda constant 7 (fold change=8.7, P=0.01), secreted phosphoprotein 24 (fold change=2.4, P=0.01) were upregulated in the drug-resistant patients. Gene ontology such as molecular functions, biological processes, and cellular components of the statistically significant differentially expressed proteins were analysed by STRINGv.11 (STRING). Gene set enrichment analysis of six statistically differentially expressed proteins were analysed using Fun Rich 3.1.3. In Biological pathway, a set of statistically differentially expressed genes (33.33%) was involved in the initial triggering of complement, synthesis, secretion, and diacylation of ghrelin, complement cascade, innate immune system, epithelial-to-mesenchymal transition, diabetes pathways, and immune system. In biological processes, a set of genes (25%) was involved in immune response, cell growth /maintenance, protein metabolism, energy pathways, and metabolism. All proteins were extracellular. Molecular function analysis of a set of genes (25%) indicates the complement activity, protease inhibitor activity, extracellular matrix structural constituent, hydrolase activity etc. All sets of genes encoded proteins in the plasma. LHX3 and HNF4A transcription factors were regulating the set of genes (100%) in the plasma of drug resistant patients. Statistically significant differentially expressed genes were analysed through the KEGG pathway (www.kegg.jp).

## Pathway list by KEGG:

[1] hsa04610: Complement and coagulation cascades - Homo sapiens (human)

[2] hsa05322: Systemic lupus erythematosus - Homo sapiens (human)

[3] hsa05133: Pertussis - Homo sapiens (human)

[4] hsa05150: Staphylococcus aureus infection - Homo sapiens (human)

## Diseases:

[1] H00080: Systemic lupus erythematosus

[2] H00102: Classic complement pathway component defects

[3] H01649: Schizophrenia

## Drug and protein interaction study:

The six-drug respondent and drug resistant patients were in first-line ART such as ZLE, ZLN, SLN, TLE, and TLN during their course of treatment in this period. The drug interactions with these proteins were analysed (www.dgidb.org) [15]. A total of 44 proteins were identified which were interacting with zidovudine, lamivudine, stavudine, nevirapine, and efavirenz drugs. Tenofovir interaction with any protein was not observed in this drug interaction database.

The serial number of the down regulated and up regulated proteins in the heat map is represented in the Gene symbol: 1.C4A 2. IGHM 3. APOA4 4.HBB, 5. SERPING1 6. PZP 7. IGHG2 8.CD5L 9.HPR 10. HBA1 11. JCHAIN 12. APOL1 13. IGHV1-2 14. IGHV3-9 15. IGHV5-51 16. C4B 17. BCHE 18. LPA 19. IGLV6-57 20. SERPINA1 21. IGKV2-24 22. IGLV3-10 23. FAT-1 24.BIN3 25. PROC 26. FN1 27. IGHA1 28. GSN 29. AFM 30. TTR 31. IGLV3-21 32. KRT1 33. IGLV3-19 34. IGKV3-15 35. C4BPB 36. FBLN1 37. IGKV1-5 38. F5 39. IGKV3-11 40. IGLV7-46 41. VNN1 42. IGLC7 43. IGHA2 44. IGHV3-13 45. IGHV1OR15-1 46. SPP2 47. CDKL3 48. IGLV10-54 49. CDC42BPA 50. SP5 51. BEX5 52. PEPD 53. IGHV6-1 54. IGHV1-45 55. CDH5 56. 3 57. SERPINA 10. The heat map of differentially expressed proteins was created by the online tool (http://www.heatmapper.ca/expression).

The gene list for a category of the molecular function of the differential expressed proteins are presented in the bar diagram. The number of genes involved in the different functional role is presented in serial number in the bar diagram 1. Binding (GO:0005488), 2. Catalytic activity (GO:0003824), 3. Molecular function regulator (GO:0098772), 4. Molecular transducer activity (GO:0060089), 5. Transcription regulator activity (GO:0140110), 6. Transporter activity (GO: 0005215). The molecular function of the differential expressed proteins was created by the online tool PANTHER- gene analyst ( http://www.pantherdb.org ).

The red colour dots represent the down-regulated proteins and black colour dots represent the up-regulated proteins (Graph pad Prism 8, USA). The volcano plot was created by Graph Pad Prism 8, USA( https://www.graphpad.com /scientific-software/prism/).

The details description of the protein-protein interaction network nodes represents proteins: Splice isoforms or post translational modifications are collapsed i.e. Each node represents all the proteins produced by a single protein-coding gene locus. The edges represent protein-protein associations: Network status: number of nodes: 35, number of edges:80, average node degree: 4.57, average local clustering coefficient:0.453, expected number of edges: 7, PPI enrichment p-value:< 1.0e-16. The protein-protein interaction network was created by using the online tool STRING Version 11.0 (https://string-db.org).

## Discussion:

The differential expression and molecular function of the Serotransferrin and Apolipoprotein A1 was identified in HIV-1 patients treated with first line ART [16]. Now, the fundamental role of these six statistically significant differentially expressed proteins such as complement C4-A, immunoglobulin heavy variable 1-2, carboxylic ester hydrolase, fibulin-1, immunoglobulin lambda constant-7, secreted phosphoprotein 24 and their association with several human diseases were discussed. The complement system is activated in the human plasma after HIV-1 infection. Once the complement is activated after infection, the outcome would be virolysis. The intricacies between complement and antibodies were reviewed during HIV infection [17]. C4 is an important component in the complement system that plays important role in defence mechanism. C4 deficiency is associated with several bacterial and viral diseases.C4a is an allotype of C4gene. C4a protein is an anaphylatoxin family of proteins produced by the activation of complement. We found, C4a protein is downregulated in drug-resistant HIV-1 infected patients. It can be considered that the down regulation of C4a can increase the viral copy number in drug-resistant HIV-1 patients. Down regulation of the C4a protein may not activate the complement system and virolysis may not happen after activation of the complement system. Even the patients are in first-line anti-retroviral therapy, the drugs would not work to kill the virus due to NRTIs and NNRTIs associated mutations in the RT gene of HIV-1. Therefore, this may be hypothesized that the RT gene mutation and downregulation of C4a have the impacts on increased viral copy number in drug-resistant HIV-1 patients. Human antibodies play an important in the control of HIV-1 infection. The strategy for boosting the early HIV-1 specific IgG response should include in early antiretroviral therapy and trials of therapeutic vaccines in HIV patients [18]. The immunoglobulin heavy chain variable region (IGHV) is responsible for antigen binding and comprised of HIV-1 individuals who developed neutralizing antibodies. The variable region of immunoglobulin heavy variable 1-2 was participated in antigen recognition. Immunoglobulins are antibodies secreted by B-lymphocytes. This variable region of immunoglobulin eliminates the bound antigen and an effector molecule in humoral immunity [19]. Thus, the down regulation of the immunoglobulin heavy variable 1-2 may affect the binding activity and elimination of the HIV particles in this drug - resistant HIV-1 patients. Carboxylic ester hydrolase plays an important role in the hydrolysis of some drugs into inactive metabolites in human plasma. The study of the role of esterase would be helpful in drug development and clinical pharmacotherapy [20]. The oral antiretrovirals first hydrolysed and then absorbed into the gastrointestinal tract. The implications of safe drug therapy are so important in HIV-1 patients. The down regulation of the carboxylic ester hydrolase may not hydrolyse the drugs in the case of these drugs - resistant HIV-1 patients. Fibulin 1 act as a tumour suppressor gene and angiogenesis inhibitor in bladder cancer. Fibulin 1 was epigenetically down regulated in bladder cancer. Fibulin-1 down regulation was associated with the non-muscle invasive bladder cancer grade and recurrence [21]. However, the fibulin-1 is up-regulated in the plasma of dug resistant HIV-1 patients by the SWATH-MS data analysis. These three-drug resistant HIV-1 patients were failure with first line ART over 1 to 6 years. The expression of fibulin-1 is probably the novel finding in drug resistant HIV-1 patients. The implication of this finding is yet to be explored. The immunoglobulin lambda chain gene was encoded by two separate germ line genes such as specificity region gene and common region gene, expressed a single continuous polypeptide chain. The main role of Immunoglobulin λ chain is antigen binding in human infection and has shown biased properties. A significant bias toward use of the λ light chain in the anti-HIV Env response was observed in individuals with acute HIV infection, those with chronic HIV infection, HIV-negative vaccines, and HIV clades (clade B, clade G, and CRF02_AG). The biased λ chain is associated with enhanced binding of anti-HIV Env glycoprotein antibodies in HIV patients and an effector molecule in humoral response [22]. The protein immunoglobulin lambda constant 7 is upregulated in drug resistant HIV-1 patients and this upregulated expression in plasma may not compete in the enhanced binding of anti-HIV Env glycoprotein and may not act as an effector molecule in humoral response in drug resistant patients. Secreted phosphoprotein is a member of cystatin superfamily encoded by SPP2 gene. Secreted phosphoprotein 24 is a bone matrix protein secreted from the liver. The secreted phosphoprotein 24 regulates bone metabolism [23]. Bone morphogenetic proteins bind to and affect the activity of bone morphogenetic proteins. SPP24 protein regulates the formation and administration of the activity of the TGF-β during bone growth and development [24]. Data shows that the secreted phosphoprotein 24 is upregulated in drug-resistant patients that may affect the bone metabolism in drug resistant HIV-1 patients. These major findings of the six novel plasma proteins in drug resistant patients will add value in the basic proteomic study by SWATH-MS. In future, studying the mechanism of these novel proteins by in vitro and in vivo experiments would throw some light in the life cycle of HIV-1 and drug metabolism in human plasma.

## Conclusion:

We are report that proteins identified in drug resistant and drug sensitive HIV-1 infected patients who are taking first line ART over 6 years by SWATH-MS. Plasma proteins such as complement C4-A, immunoglobulin heavy variable 1-2, carboxylic ester hydrolase, fibulin-1, immunoglobulin lambda constant7, secreted phosphor protein 24 were differentially expressed in individual drug resistant HIV-1 subtype C patients. The role of these proteins is reported using gene ontology analysis. This study would be helpful for further implementation in the search for biomarker discovery in HIV-1/AIDS patients taking first line ART.

## Financial support and sponsorship:

The project "Characterization of drug resistant HIV-1 mutants of Agra region, India by genomic and proteomic approaches" funded for senior research fellowship of Sushanta Kumar Barik (File No. 80/990/2015-ECD-I). M. M. Alam, JALMA, ICMR and Rakesh Kumar Mishra, ART centre, S.N Medical College are acknowledged for providing technical help in the collection of blood samples.
